# The Hypernetted Chain Equations for Periodic Systems

**DOI:** 10.1007/s10909-017-1771-5

**Published:** 2017-03-20

**Authors:** Martin Panholzer

**Affiliations:** Laboratoire des Solides Irradies, Ecole Polytechnique, CNRS-CEA, Universite Paris-Saclay, 91128 Palaiseau cedex, France

**Keywords:** HNC, Periodic systems, Reciprocal space

## Abstract

Starting from the general inhomogeneous Fermi hypernetted chain equations, the equations for periodic systems are derived by simple Fourier transform. It is shown how the symmetry reduces the size of the involved quantities. First results for a one-dimensional (1D) model system are presented. The results allow a reliable estimation of the numerical demand even for realistic 3D systems, such as solids. It is shown that treatment of this systems is feasible with moderate computational resources.

## Introduction

Jastrow correlated trial wave functions, i.e., a wave function which additionally to a Slater determinant includes two-particle correlations, are widely used in quantum Monte Carlo (MC) techniques [1, 2]. Less well known are the analytic methods to calculate expectation values with this wave function [3–6]. The Fermi hypernetted chain method (FHNC) is an effective scheme to calculate a large class of cluster diagrams. The optimal correlation function is obtained by minimization of the energy expectation value. A drawback of the method, compared to MC techniques, is that certain diagrams, i.e., the elementary diagrams, are not covered by the scheme. Therefore, the obtained quantities and matrix elements are only approximations. However, the advantage of the method is that it is generally numerically less demanding than MC methods. The analytic representation of the wave functions further allows to deal with excited states [7, 8].

An additional advantage of the used formulation of the FHNC method is that the functional form of the method allows a parameter-free optimization of the two-particle correlation function.

The FHNC method has been first developed for homogeneous systems, but has been generalized to inhomogeneous systems by Krotscheck et al. [9–12]. In order to keep the numerical demand low, one has to utilize the symmetries of the system. This has been done for slab [13] and for spherical geometries.

In this paper a possible discretization for periodic systems is presented which reduces the numerical demand considerably and leads to a simple form of the Euler equation. A different discretization and coordinates are proposed by Krotscheck [14]. Although it allows a further reduction in numerical demand by reducing the resolution of the center of mass coordinate, it sacrifices the simplicity of the relevant equations and reduces the resolution and consequently the accuracy of the result.

This work is related to an implementation of Fantoni and Schmidt [15], which formulates and solves the FHNC equations in a periodic box, but with a uniform density. The presented method extends this approach by explicitly including a non-constant density.

## The Inhomogeneous HNC Equations

Here the generalization to the general inhomogeneous case of the FHNC equations in the formulation of Kallio and Piilo [16] is given. The reason for this is that results are shown for a 1D model system and the simplified FHNC of Krotscheck doesn’t work in 1D.

The inhomogeneous theory yields coupled equations for the one-particle and two-particle correlations. The one-particle equation is a generalized Hartree–Fock equation (gHF) as given in [12], and the two-particle equation is actually a set of equations given below. These two equations are coupled and need to be solved self-consistently. However, it seems to be a reasonable approximation for certain systems to omit the coupling [13]. This is the justification for using a model for the single-particle states in the next section and not solving the gHF.

The inhomogeneous Euler–Lagrange equations can be written as

1 $$\begin{aligned} \left[ S^{-1} *H_1 *S^{-1} \right] ({\mathbf {r}}_{\mathbf {1}},{\mathbf {r}}_{\mathbf {2}})=2V_\mathrm{ph}({\mathbf {r}}_{\mathbf {1}},{\mathbf {r}}_{\mathbf {2}}) + H_1 ({\mathbf {r}}_{\mathbf {1}},{\mathbf {r}}_{\mathbf {2}}) \end{aligned}$$

with the definition of the convolution

2 $$\begin{aligned}{}[A*B]({\mathbf {r}}_{\mathbf {1}},{\mathbf {r}}_{\mathbf {3}})= \int {d^3}{r_2} A({\mathbf {r}}_{\mathbf {1}},{\mathbf {r}}_{\mathbf {2}})B({\mathbf {r}}_{\mathbf {2}},{\mathbf {r}}_{\mathbf {3}}) \end{aligned}$$

and where *S* is the, to be determined, static structure function. $V_\mathrm{ph}$ is the particle hole irreducible interaction explicitly given below. The one-body operator $H_1$ is defined as3where $\rho _1$ is the one-body density obtained from the gHF.

The induced interaction is

4 $$\begin{aligned} w_{\mathrm {I}}({\mathbf {r}}_{\mathbf {1}},{\mathbf {r}}_{\mathbf {2}})=-\frac{\left[ V_\mathrm{ph}({\mathbf {r}}_{\mathbf {1}},{\mathbf {r}}_{\mathbf {2}})+\frac{1}{2} (S *H_1 + H_1 *S )-H_1 \right] ({\mathbf {r}}_{\mathbf {1}},{\mathbf {r}}_{\mathbf {2}})}{\sqrt{\rho _1({\mathbf {r}}_{\mathbf {1}})\rho _1({\mathbf {r}}_{\mathbf {2}})}} \; , \end{aligned}$$

where the $V_\mathrm{ph}$ from the previous iteration is used. (In order to start the iteration process, a reasonable guess for $V_\mathrm{ph}$ is needed.)

With these ingredients and by using the pair distribution function $g({\mathbf {r}}_{\mathbf {1}},{\mathbf {r}}_{\mathbf {2}})$, the new particle-hole irreducible interaction is computed5where $\nabla _1$ is short for the gradient with respect to ${\mathbf {r}}_{\mathbf {1}}$ and *v* is the interaction potential of the particles in the system. Note that here no convolutions are involved, i.e., the functions are simply multiplied. The Fermi potential $v_\mathrm{F}$ is defined as6with

7 $$\begin{aligned} w_\mathrm{IF}({\mathbf {r}}_{\mathbf {1}},{\mathbf {r}}_{\mathbf {2}})=-\frac{\left[ S_\mathrm{F}^{-1} *H_1 *S_\mathrm{F}^{-1} + S_\mathrm{F} *H_1 + H_1 *S_\mathrm{F}- 3 H_1 \right] ({\mathbf {r}}_{\mathbf {1}},{\mathbf {r}}_{\mathbf {2}})}{2 \sqrt{\rho _1({\mathbf {r}}_{\mathbf {1}})\rho _1({\mathbf {r}}_{\mathbf {2}})}} \;. \end{aligned}$$

The free static structure function $S_\mathrm{F}$ is given by

8 $$\begin{aligned} S_\mathrm{F}({\mathbf {r}}_{\mathbf {1}},{\mathbf {r}}_{\mathbf {2}})=\delta ({\mathbf {r}}_{\mathbf {1}}, {\mathbf {r}}_{\mathbf {2}})-\frac{1}{\nu }\frac{|l({\mathbf {r}}_{\mathbf {1}},{\mathbf {r}}_{\mathbf {2}}) |^2}{\sqrt{\rho ({\mathbf {r}}_{\mathbf {1}})\rho ({\mathbf {r}}_{\mathbf {2}})}} \end{aligned}$$

where *l* is the non-interacting density matrix as result of the gHF and $\nu$ is the spin degeneracy and $g_\mathrm{F}$ is the free pair distribution function analog of $S_\mathrm{F}$.

In order to obtain the boson version of the equations $v_\mathrm{F}$ has to be set to zero.

**Solution of the Euler equation:** Equation (1) is solved by considering the eigenvalue problem

9 $$\begin{aligned} \int d^3 r_2 \left[ 2V_{p-h} + H_1 \right] ({\mathbf {r}}_{\mathbf {1}},{\mathbf {r}}_{\mathbf {2}}) H_1 \psi _l({\mathbf {r}}_{\mathbf {2}})=\hbar ^{2}\omega _{l}^{2} \psi _{l}(\mathbf {r}_\mathbf {1}) \end{aligned}$$

The $\psi _l$ are orthonormalized according to

10 $$\begin{aligned} \int d^3r \psi _i({\mathbf {r}}) H_1({\mathbf {r}}) \psi _j({\mathbf {r}})=\delta _{ij} \end{aligned}$$

The static structure factor is obtained by11and its inverse12


## The Equations for the Periodic System and Results for a 1D Model

### Definition of the Fourier Transform

In order to describe a periodic system a unit cell, spanned by the lattice vectors ${\mathbf {a}}_{\mathbf {1}}\cdots {\mathbf {a}}_{\mathbf {d}}$, where *d* is the dimensionality of the system, is defined. One-body quantities like the density are periodic: $\rho ({\mathbf {r}}+{\mathbf {T}}_{\mathbf {n}})=\rho ({\mathbf {r}})$. With the translation vector ${\mathbf {T}}_{\mathbf {n}}=n_1{\mathbf {a}}_{\mathbf {1}}\cdots +n_d{\mathbf {a}}_{\mathbf {d}}$. A similar relation also holds for two-body quantities, e.g., the pair distribution function: $g({\mathbf {r}}_{\mathbf {1}}+{\mathbf {T}}_{\mathbf {n}},{\mathbf {r}}_{\mathbf {2}}+{\mathbf {T}}_{\mathbf {n}}) =g({\mathbf {r}}_{\mathbf {1}},{\mathbf {r}}_{\mathbf {2}})$. This symmetry is used to constrain the first coordinate to the basic unit cell. This is denoted by a bar: $g(\bar{{\mathbf {r}}}_{\mathbf {1}},{\mathbf {r}}_{\mathbf {2}})$. Further, we may decompose the second coordinate: $g(\bar{{\mathbf {r}}}_{\mathbf {1}},\bar{{\mathbf {r}}}_{\mathbf {2}}+{\mathbf {T}}_{\mathbf {n}})$. In order to describe pair properties properly we have to choose a cutoff in the translations. Therefore, we restrict the integers $n_i$ to the interval $0\cdots N-1$,^1^ which we call now crystal on which we impose periodic boundary conditions. (Although this is non-physical, it becomes a good approximation if *N* is large enough.) This crystal consists of $N^d$ unit cells. The unit cell is discretized with $n_G$ points in each direction, so the resolution is $\Delta r_i=\frac{a_i}{n_G}$. A periodic function can be expressed as a sum of plane waves

13 $$\begin{aligned} f({\mathbf {r}})=\sum _{{\mathbf {G}}} f_{{\mathbf {G}}} e^{i{\mathbf {Gr}}} \end{aligned}$$

where ${\mathbf {G}} = m_1{\mathbf {b}}_{\mathbf {1}}\cdots +m_d{\mathbf {b}}_{\mathbf {d}}$ are the reciprocal lattice vectors, with ${\mathbf {a}}_{\mathbf {i}}{\mathbf {b}}_{\mathbf {j}}=2\pi \delta _{i,j}$. For the discretized function in $f({\mathbf {r}}=\sum _{i=1}^d l_i \frac{{\mathbf {a}}_i}{n_G})$ with $0\leqq l_i < n_G$ integer, the reciprocal lattice sum (13) is limited by $n_G$.

By utilizing the periodicity of the system the Fourier transform is defined as

14 $$\begin{aligned} f({\mathbf {q}},{\mathbf {G}},{\mathbf {G}}') =\frac{1}{V^2_\mathrm{Crystal}}\int d^3r_1d^3r_2e^{i({\mathbf {q}}+{\mathbf {G}}){\mathbf {r}}_{\mathbf {1}}} e^{-i({\mathbf {q}}+{\mathbf {G}}'){\mathbf {r}}_{\mathbf {2}}}f({\mathbf {r}}_{\mathbf {1}},{\mathbf {r}}_{\mathbf {2}}) \end{aligned}$$

and its inverse

15 $$\begin{aligned} f({\mathbf { r}}_{\mathbf {1}},{\mathbf {r}}_{\mathbf {2}})= \sum _{{\mathbf {q}},{\mathbf {G}},{\mathbf {G}}'}e^{-i({\mathbf {q}}+{\mathbf {G}}) {\mathbf {r}}_{\mathbf {1}}}e^{i({\mathbf {q}}+{\mathbf {G}}') {\mathbf {r}}_{\mathbf {2}}} f({\mathbf {q}},{\mathbf {G}},{\mathbf {G}}') \end{aligned}$$

With this definition the convolution as defined above becomes in momentum space

16 $$\begin{aligned} \mathcal{FT} \left[ [A*B]({\mathbf {r}}_{\mathbf {1}},{\mathbf {r}}_{\mathbf {3}})\right] ({\mathbf {q}}, {\mathbf {G}}_{\mathbf {1}},{\mathbf {G}}_{\mathbf {3}})=\,&V_{\mathrm{Crystal}}\sum _{{\mathbf {G}}_{\mathbf {2}}}A({\mathbf {q}},{\mathbf {G}}_{\mathbf {1}},{\mathbf {G}}_{\mathbf {2}}) B({\mathbf {q}},{\mathbf {G}}_{\mathbf {2}}, {\mathbf {G}}_{\mathbf {3}}) \nonumber \\ \equiv \,&[A*B]({\mathbf {q}},{\mathbf {G}}_{\mathbf {1}},{\mathbf {G}}_{\mathbf {3}}) \end{aligned}$$

With the above definitions the Euler equation is written in reciprocal space as

17 $$\begin{aligned} \left[ S^{-1} *H_1 *S^{-1} \right] ({\mathbf {q}},{\mathbf {G}}_{\mathbf {1}},{\mathbf {G}}_{\mathbf {2}})=[2V_\mathrm{ph} + H_1] ({\mathbf {q}},{\mathbf {G}}_{\mathbf {1}},{\mathbf {G}}_{\mathbf {2}}) \end{aligned}$$

The induced interaction is also calculated most effectively in reciprocal space, by Fourier transform or Eq. (4). However, the particle hole irreducible interaction is calculated in r-space, like in the homogeneous case.

The most time-consuming part is the solution of the Euler equation. The Fourier transform reduces the eigenvalue problem Eq. (9) considerably since ${\mathbf {q}}$ appears as a parameter:18so the computation timescales are linear with the number of *q*-vectors.

### Results for a 1D Model System

The described method is applied to a 1D model system similar to that described by Asgari [17]. The interaction potential is derived from the electron gas in a homogeneous trap $v(r)=\frac{e^2 \sqrt{2}}{2b}\exp {\frac{r^2}{4b^2}} \; \mathrm {erfc}\frac{|r|}{2b}$ where *b* characterizes the thickness of the wire. Since the single-particle equation is not solved, a simple model for the single-particle basis is used

19 $$\begin{aligned} \sqrt{V_{\mathrm{Crystal}}} \phi _k (r)=\sqrt{1-2\lambda ^2} e^{ikr} +\lambda e^{i(k+G)r}+\lambda e^{i(k-G)r} \;. \end{aligned}$$

These single-particle states result in a sinusoidal modulated density with the amplitude determined by $\lambda$. $G=\frac{2\pi }{a}$ is the reciprocal lattice vector and *a* the length of the unit cell.

**Fig. 1:**
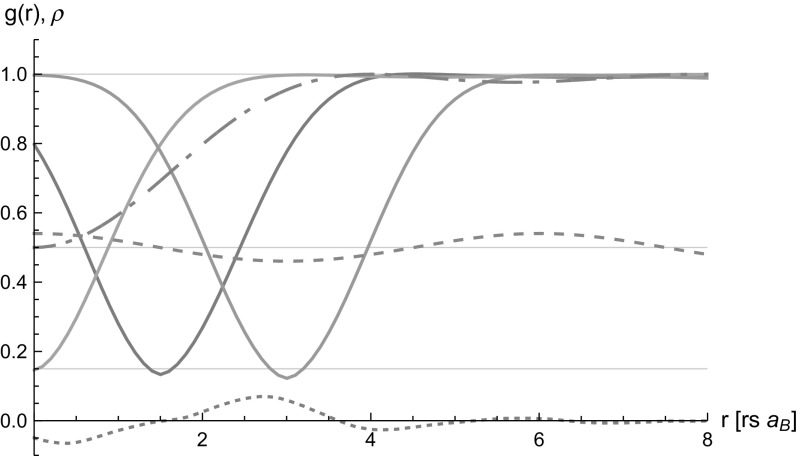
Pair distribution function of an inhomogeneous system is shown at different points *g*(0, *r*), *g*(1.5, *r*) and *g*(3, *r*) (*solid lines*, distinguished by the minimum). Also shown are the density (*dashed*), the non-interacting pair distribution function $g_\mathrm{F}(0,r)$ (*dashed dotted*) and the difference of the inhomogeneous and homogeneous result $5 \cdot (g_{\mathrm{hom}}(r-1.5)-g(1.5,r))$ (*short dashed*)

Results of this model are plotted in Fig. 1 with the parameters $a=6r_S a_B, b=0.1r_S a_B, \lambda =0.02$ and the Wigner–Seitz radius $r_S=1$ and $a_B=0.529$Å the Bohr radius. In order to converge the result a real space resolution of $\Delta r=0.1r_Sa_B$ was sufficient for the homogeneous and the inhomogeneous implementation. This results in $n_G=60$ reciprocal lattice points, and the number of *q*-points needed is $n_q=20$ which determine the size of the large cell. It has been verified that the implementation reproduces the FHNC/0 result of [17] in the homogeneous limit. In Fig. 1 the deviation of the inhomogeneous pair distribution function from the homogeneous on is clearly seen. Further, the minimum in $g(r,r')$ at $r=r'$ follows approximately a local density approximation.

The number of *G* vectors $n_G^3$ for a realistic 3D system is now estimated. Sodium is used as an example. The lattice constant of the primitive unit cell is $a=1.75r_s a_B$, where $r_s=4$ for sodium. From the treatment of the homogeneous electron gas and the results for the 1D model system it is seen that $n_G=20$ points are sufficient for that length. It is even possible to reduce this number further. This results in a $8000\times 8000$ eigenvalue problem for each *q*-vector, which is numerically tractable. Only *q*-vectors in the irreducible Brillouin zone need to be calculated, which further reduces the numerical load.

## Conclusions

The FHNC equations for periodic systems have been derived by simple Fourier transform of the general inhomogeneous equations. This considerably reduces the numerical demand so that realistic systems become numerically feasible. Further reduction in the numerical demand is possible, but only at the cost of simplicity of the theory.

First results for a 1D system have been shown. There is increasing interest in the theoretical description of 1D systems, e.g., [18–20]. The presented method could extend existing methods by applying it to more realistic systems. Also more fundamental questions could be addressed, like the extension of the local density approximation to pair quantities could be investigated. That is how to combine results of the homogeneous system for different densities to obtain an estimation of the inhomogeneous result. Work in that direction is in progress.

## Footnotes

1 For simplicity the same *N* is used for every direction, and a generalization to different *N* for each direction is easily possible. The same is done for $n_G$.
